# Maximizing Network Resilience against Malicious Attacks

**DOI:** 10.1038/s41598-019-38781-7

**Published:** 2019-02-19

**Authors:** Wenguo Li, Yong Li, Yi Tan, Yijia Cao, Chun Chen, Ye Cai, Kwang Y. Lee, Michael Pecht

**Affiliations:** 1 College of Electrical and Information Engineering, Hunan University, Changsha, 410082 China; 2 School of Information and Electronic Engineering, Hunan City University, Yiyang, 413000 China; 3 School of Electrical and Information Engineering, Changsha University of Science and Technology, Changsha, 410114 China; 4 Department of Electrical and Computer Engineering, Baylor University, Waco, Texas 76798-7356 USA; 5 CALCE Electronics Products and Systems Center, University of Maryland, College Park, Maryland 20742 USA

## Abstract

The threat of a malicious attack is one of the major security problems in complex networks. Resilience is the system-level self-adjusting ability of a complex network to retain its basic functionality and recover rapidly from major disruptions. Despite numerous heuristic enhancement methods, there is a research gap in maximizing network resilience: current heuristic methods are designed to immunize vital nodes or modify a network to a specific onion-like structure and cannot maximize resilience theoretically via network structure. Here we map complex networks onto a physical elastic system to introduce indices of network resilience, and propose a unified theoretical framework and general approach, which can address the optimal problem of network resilience by slightly modifying network structures (i.e., by adding a set of structural edges). We demonstrate the high efficiency of this approach on three realistic networks as well as two artificial random networks. Case studies show that the proposed approach can maximize the resilience of complex networks while maintaining their topological functionality. This approach helps to unveil hitherto hidden functions of some inconspicuous components, which in turn, can be used to guide the design of resilient systems, offer an effective and efficient approach for mitigating malicious attacks, and furnish self-healing to reconstruct failed infrastructure systems.

## Introduction

Maximizing network resilience is of great importance because it helps to mitigate the impact of perturbations or failures and suggests an emergency solution to repair the network^1–4^. Recently, considerable research effort has been devoted to enhancing network resilience against malicious attacks^5–25^, including immunization strategies^5,6,10–16^ and topological construction methods^17–25^. Most of the immunization strategies map the problem onto the identification of vital nodes, which, if immunized, would mitigate the diffusion of a large scale failure. However, the strategies cannot essentially improve network resilience from a topological structure, and it is impossible to find a universal index to quantify the importance of a node well in every situation^16^.

The problem of maximizing network resilience with topological construction is to find an optimal set of edge swaps (or edge additions). The heuristic edge-swap (ES) methods^17–21^ can enhance network resilience by modifying a network to a specific onion-like structure. However, the computations of these methods become prohibitively expensive, especially for the large scale networks; on the other hand, the networks optimized by the ES methods have a great change in topological structures (onion-like structures), which has an impact on the functionality of the original networks. In the heuristic edge-addition (EA) methods^22–25^, for a given network, the new edges between the nodes with lowest degrees are added into the original network. The EA methods have a good performance on computational complexity; however, they possess few effect on resilience optimizations. Furthermore, both the ES and the EA methods cannot optimize network resilience globally. As a consequence, they cannot well maintain the topological functionality of a network and their performance on resilience improvement cannot be guaranteed.

Measurement of resilience is essential for addressing the resilience optimization problem, yet there are no universally accepted indices of network resilience. Conventionally, the resilience (or robustness) of networks is measured by critical (percolation) threshold^2–6^ which is equivalent to the maximum external force in physical elastic systems. Hence, the measurement cannot fully characterize the elastic properties of nonlinear networks (see also Fig. S1). Ref.^17^ defined a robustness measurement *R*, but without mathematical deductive inference and physical properties. Other defined resilience metrics^7,8^ vary between extremes such as recoverability, adaptability and absorptivity^9^. Therefore, the problem of maximizing network resilience remains unsolved despite an abundance of heuristic methods^17–25^. More efforts are required for a general approach to maximize network resilience.

Here we address the problem of optimal resilience by finding an optimal (that is, minimal) set of structural edges. After introducing network resilience indices that can reflect the most essential resilience properties of network structure, we provide an optimal solution of the problem by means of a unified theoretical framework and the proposed indices. Further, we propose an algorithm of posteriorly adding (PA) edges to solve the resilience-optimization problem in artificial random networks and real networks^26–28^. Compared with competing approaches^17,23^, our algorithm achieves better network resilience performance. The main contributions of this paper are as follows: (1) by mapping a complex network onto a physical elastic system, we introduce indices of network resilience, which can better characterize the elastic properties for nonlinear networks, compared with the conventional metrics; (2) based on the proposed indices, we present a unified theoretical framework and a PA algorithm, which can maximize network resilience with minimal costs (i.e., with an optimal (that is, minimal) set of structural edges), in contrast to the heuristic ES^17^ and EA^23^ methods.

## Methods

### Resilience indices

The resilience metrics of networks in this paper were formulated by mapping a complex network onto a physical elastic system and can be commonly used in complex networks. For a physical elastic system, resilience is defined as the capacity of a material to absorb energy during elastic deformation, which can be measured by elastic potential energy (elastic strain energy), i.e.,

1 $${E}_{p}={\int }_{\sigma =0}^{{\sigma }_{c}}-F{\rm{d}}\sigma $$

where *F* is external force (stress), *σ* is elastic deformation and *σ*_*c*_ is critical elastic deformation. For a linear physical elastic system, the external force (or elastic deformation) is also a resilience metric, and it has the identity with the elastic potential energy. The value of *Ep* of Equations (1) lies in the range [0, ∞).

In analogy with the physical elastic system, the proposed network resilience refers to the network deformation under external force including initial attacks, disruptions or perturbations. Let the fraction of the removed nodes, *q*, represent external force; and let the fraction of failed nodes, 1 − *G*(*q*), denote elastic deformation under external force, where *G*(*q*) is the fraction of the largest (giant) connected component^3,12,29^. And support that the size and shape of a complex network can be restored during elastic deformation if the external force is withdrawn. The elastic potential energy of a complex network, *E*_*p*_, can be given by (see also Fig. S1a, and detailed explication in Supplementary Information Section S1).

2 $${E}_{p}={\int }_{{\rm{1}}-G(q)=0}^{{\rm{1}}-G(q)={\rm{1}}}-q{\rm{d}}(1-G(q))={\int }_{G(q)=0}^{G(q)={\rm{1}}}q{\rm{d}}G(q)={\int }_{q=0}^{q=1}G(q)){\rm{d}}q$$

where *q* ϵ [0, 1], *G*(*q*) ϵ [0, 1] and 1 − *G*(*q*) ϵ [0, 1]. If *q* = 0 (the network is not attacked), *G*(*q*) = 1 and 1 − *G*(*q*) = 0; If *q* > *q*_*c*_ (the network breaks down), *G*(*q*) = 0 and 1 − *G*(*q*) = 1 − *G*(*q*_*c*_) = 1, where *q*_*c*_ is the critical external force (the critical threshold) and *G*(*q*_*c*_) is the critical giant connected component^29^. The value of *E*_*p*_ of Equations (2) E_p_ lies strictly in the range [0, 0.5].

Considering that the network system is a nonlinear discrete-time system, the quadrature formula (2) can but be solved by numerical integration method, here we provide the numerical versions of Equation (2) by rectangular and trapezoid approximation methods respectively

3 $${E}_{p}=\frac{1}{N}{\sum }_{q=\frac{1}{N}}^{1}G(q)$$

4 $${E}_{p}=\frac{1}{N}{\sum }_{{q}_{l}=\frac{1}{N}}^{1}\frac{G({q}_{l})+G({q}_{l-1})}{2}$$

where *N* is the total number of nodes in the network, 1/*N* is the normalized minimum-step integral size which corresponds to d*q* in the Equation (2), and *q*, *q*_*l*_ and *q*_*l*−1_ are the fractions of the removed nodes and *q*_*l*_ − *q*_*l*−1_ = 1/*N*. The value of *E*_*p*_ of Equations (3) and (4) E_p_ lies strictly in the range [1/*N*, 0.5], where the two limits correspond to a star network and a fully connected graph respectively. This is because (1) a star network breaks down if a vital node is removed from it, and (2) if a fully connected graph is attacked maliciously (or randomly), its fraction of the largest (giant) connected component is equal to 1 minus the fraction of the attacked nodes, i.e., *G*(*q*) = 1 − *q*. Though the error of numerical integration in Equation (4) is smaller than that in Equation (3) (see the detailed explication in Supplementary Information Section S1), we select the Equation (3) as numerical integration version of Equation (2) in the following simulations in Result Section, for comparing with the method in ref.^17^. Note that in ref.^17^, the right side of Equation (3) is defined only as a robustness measure *R* without mathematical deductive inference and physical properties.

Beyond that, the complex networks have other resilient indices such as an elastic coefficient (also called the modulus of elasticity), the critical external force (critical threshold, *q*_*c*_) and the elastic complementary energy (all of which are defined in Supplementary Information Section S1), the same as the physical elastic systems do. The traditional measurement for resilience of networks, critical threshold (*q*_*c*_), can just reflect the critical external force, which is unsuitable for nonlinear systems. For a nonlinear network, the elastic potential (or complementary) energy can better characterize its elastic properties due to its advantages covering the elastic coefficient and critical threshold (see Fig. S1b, and detailed explication in Supplementary Information Section S1).

### Theoretical framework

**Figure 1:**
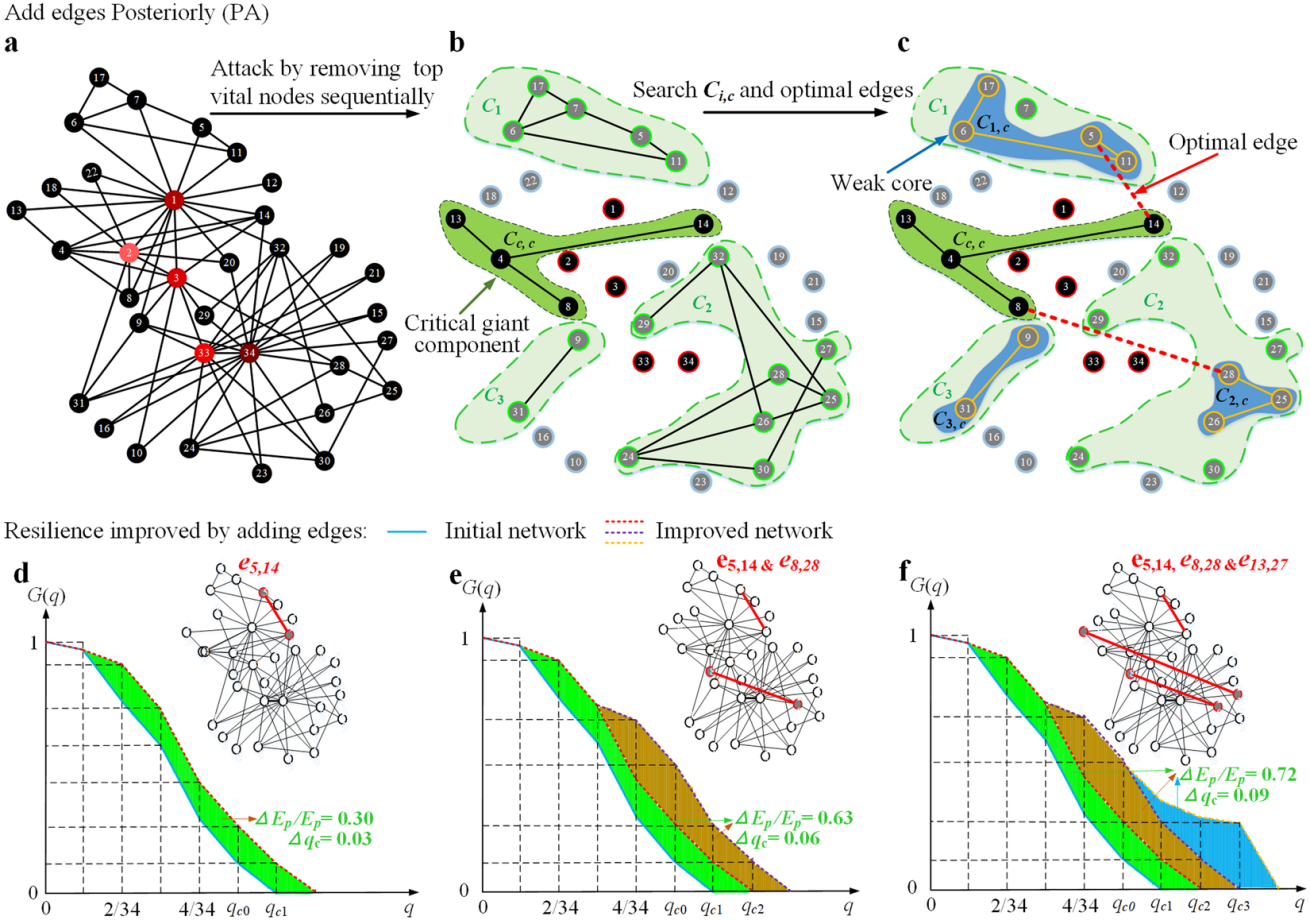
Optimal edges and weak cores: (**a**) an original network (Zachary network^26^). (**b**) The collapsed network including the isolated nodes, the finite components *C*_*i*_ and the critical giant component *C*_*c*,*c*_, by sequentially removing top vital nodes (34, 1, 3, 33 and 2). The size and sequence of finite components (*s*_*i*_, *q*_*i*_) and the critical threshold (*q*_*c*_) have been conserved in the process of malicious attacks. (**c**) The search of critical finite components (*C*_*i*,*c*_, “weak cores”) and optimal edges. The optimal set of edges including *e*_5,14_, *e*_8,28_ and *e*_13,27_ is identified, where the optimal edges are adaptively connected between the least influencer in the critical finite component with the top latent resilience and the critical giant component. (**d**–**f**) The ratios of increments of resilience and critical threshold by adding one, two and three of the optimal edges *e*_5,14_, *e*_8,28_, *e*_13,27_, respectively, where Δ*q*_*c*_ = *q*_*cI*_ − *q*_*c*_.

If a certain fraction (*q*) of vital nodes is intentionally removed from a network and the network breaks down into many finite (disconnected) components, i.e., *q* = *q*_*c*_, the network will undergo a structural collapse and no giant connected component will exist, i.e., *G*(*q*_*c*_) = 0. Let the vector ***C*** = (*C*_1_, …, *C*_*k*_, …, *C*_*K*_) represent the finite components, whose normalized sizes are *s*_1_, …, *s*_*k*_, …, *s*_*K*_ (*s*_1_ > … > *s*_*k*_ > … > *s*_*K*_), where *k* is the serial number of a finite component ordered by size, and *K* is the number of finite components in the collapsed network. Similar to the definition of the critical giant components, we define the “weak cores” (e.g., *C*_1,*c*_, *C*_2,*c*_ in Fig. 1) as the critical finite components. A critical finite component is a special critical giant component caused by an attack, as a finite component is regarded as a subnet. If an edge between the “weak cores” and the critical giant component (*C*_*c*,*c*_ in Fig. 1) is added, the failure of the finite component can be avoided unless the critical giant component *G*(*p*_*c*_) fails. Therefore, the weak cores can be used for maximizing network resilience.

For simplicity, we investigated the case of adding only one optimal edge, *e*_*ij*_, to maximize the network resilience (elastic potential energy); an optimal set of edges is provided in the follow-up section. There are only 4 ways to add this edge *e*_*ij*_: (i) in the same finite component *C*_*k*_ (*i*, *j* ϵ *C*_*k*_, *s*_*k*_ > 2/*N*), (ii) in the same critical giant component *C*_*c*,*c*_ (*i*, *j* ϵ *C*_*c*,*c*_), (iii) between two different finite components *C*_*a*_, *C*_*b*_ (*i* ϵ *C*_*a*_, *j* ϵ *C*_*b*_, *s*_*a*_ > *s*_*b*_), where *s*_*a*_ and *s*_*b*_ are the sizes of *C*_*a*_ and *C*_*b*_, respectively, and (iv) between a finite component *C*_*a*_ (*i* ϵ *C*_*a*_) and the critical giant component *C*_*c*,*c*_ (*j* ϵ *C*_*c*,*c*_). After adding an edge in any of the above 4 ways, from Equation (2), the increment of elastic potential energy of network can be given by

5 $$\Delta {E}_{p}={\int }_{q=0}^{q=1}[{G}_{I}(q)-{G}_{O}(q)]{\rm{d}}q$$

where *G*_*I*_(*q*) and *G*_*O*_(*q*) are the elastic potential energies of the modified network by adding the edge *e*_*ij*_ and the original network respectively, and Δ*E*_*p*_ ϵ [0, 0.5). Note that the least important nodes in the “weak cores” (*C*_*i*,*c*_) and the critical giant components should be selected as the terminal nodes of edge *e*_*ij*_ to avoid being attacked maliciously in cases (iii) and (iv).

**Figure 2:**
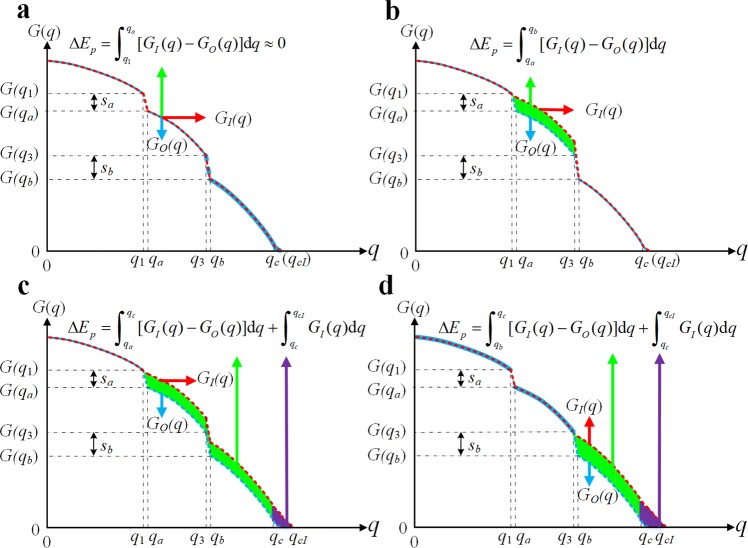
Comparisons of increments of elastic potential energy by adding edges in 4 possible ways. Here, *q*_1_ = *q*_*a*_ − 1/*N*, *q*_3_ = *q*_*b*_ − 1/*N*. (**a**) The increment of elastic potential energy by adding an edge between the two different nodes in the same finite component *C*_*a*_ (case (i)) (or in the critical giant component *C*_*c*,*c*_ (case (ii))). (**b**) The increment of elastic potential energy by adding an edge between the two nodes from two different finite components (case (iii)). (**c**) The increment of elastic potential energy by adding an edge between the “weak core” *C*_*a*,*c*_ (or *C*_*b*,*c*_ in **d**) and the critical giant component *C*_*c*,*c*_ (case (iv)).

For cases (i) and (ii), due to ${G}_{I}(q)\approx {G}_{O}(q)$ and ${q}_{a}-{q}_{1}=1/N$ (where *q*_*a*_ and *q*_1_ are the fractions of the removed nodes) (see Fig. 2a), the increment of elastic potential energy can be obtain from Equation (5) by

6 $$\Delta {E}_{p}^{{\rm{i}},{\rm{ii}}}={\int }_{q=0}^{q=1}[{G}_{I}(q)-{G}_{O}(q)]{\rm{d}}q={\int }_{{q}_{1}}^{{q}_{a}}[{G}_{I}(q)-{G}_{O}(q)]{\rm{d}}q\approx 0$$

Suppose that the two finite components *C*_*a*_ and *C*_*b*_ fail at *q*_*a*_ and *q*_*b*_ (*q*_*a*_ < *q*_*b*_) respectively, where *q*_*b*_ is the fraction of the removed nodes, and *C*_*a*,*c*_ and *C*_*b*,*c*_ are the corresponding “weak cores” of *C*_*a*_ and *C*_*b*_ respectively. Accordingly, the increment of elastic potential energies in case (iii) and (iv) are respectively given by (see Fig. 2b,c)

7 $$\Delta {E}_{p}^{{\rm{iii}}}={\int }_{q=0}^{q=1}[{G}_{I}(q)-{G}_{O}(q)]{\rm{d}}q={\int }_{{q}_{a}}^{{q}_{b}}[{G}_{I}(q)-{G}_{O}(q)]{\rm{d}}q$$

8 $$\Delta {E}_{p}^{{\rm{iv}}}={\int }_{q=0}^{q=1}[{G}_{I}(q)-{G}_{O}(q)]{\rm{d}}q={\int }_{{q}_{a}}^{{q}_{c}}[{G}_{I}(q)-{G}_{O}(q)]{\rm{d}}q+{\int }_{{q}_{c}}^{{q}_{cI}}{G}_{I}(q)$$

where *q*_*cI*_ is the critical threshold of the modified network, ${\rm{\Delta }}{E}_{p}^{{\rm{iii}}}\in [0,0.5)$ and ${\rm{\Delta }}{E}_{p}^{{\rm{iv}}}\in [0,0.5)$. Comparing Equation (8) (case (iv), Fig. 2c) with Equation (7) (case (iii), Fig. 2b), one can see that the increment of elastic potential energy in case (iv) is greater than that in case (iii), due to *q*_*c*_ > *q*_*b*_.

### Algorithm

The above analysis shows that the optimal edge, *e*_*ij*_, must be located between a “weak core” and the critical giant component (i.e., case (iv)) (strict theoretical proof in Supplementary Information Section S4). Moreover, from Equation (8), it can be observed that increment of the elastic potential energy in case (iv) depends on two key factors: the size and the failed sequence (such as *q*_*a*_ in Fig. 2c and *q*_*b*_ in Fig. 2d) of the finite component. Greater finite component size and smaller failed sequence result in greater increment of elastic potential energy, as shown in Fig. 2c,d.

**Figure 3:**
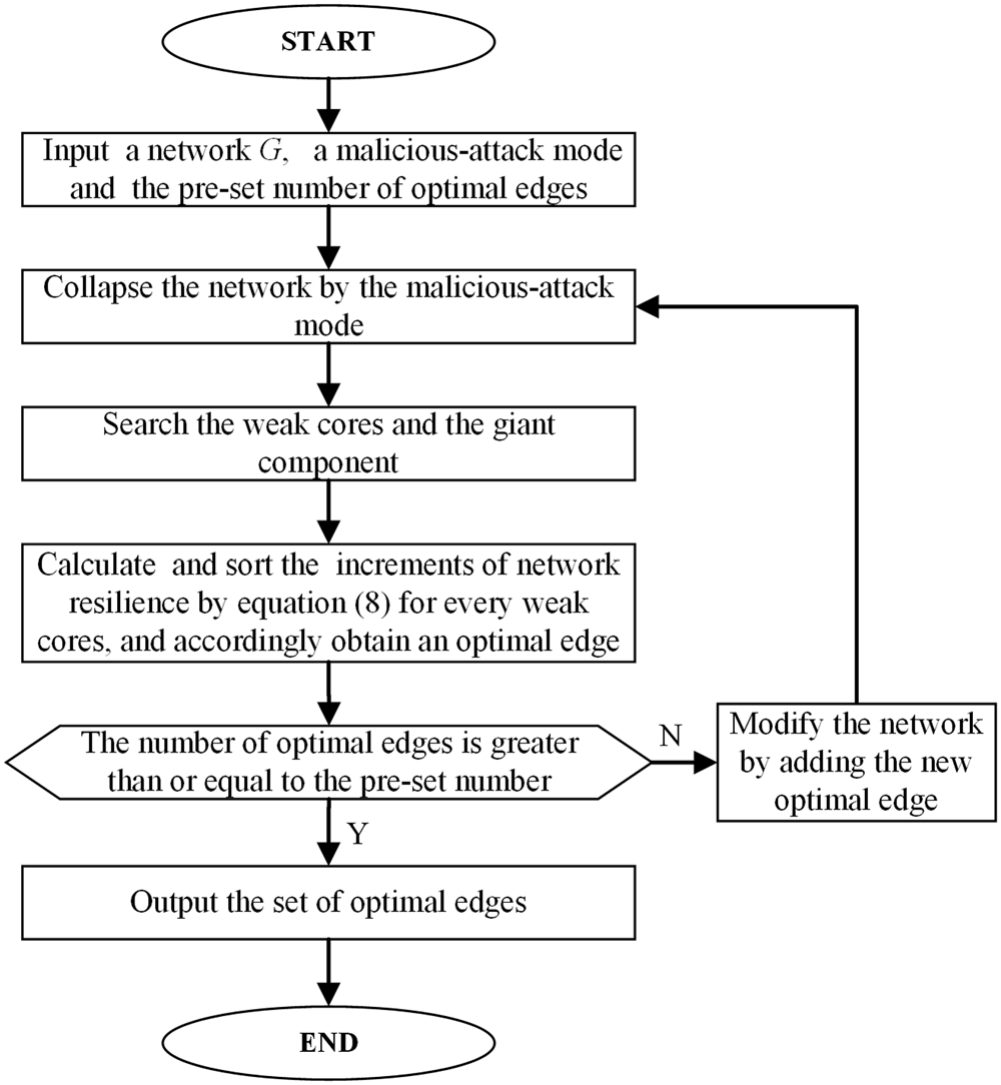
The overall flowchart of the proposed PA algorithm.

By comparing the increment of the elastic potential energy from Equation (8) for every finite component, the sequence of the set of increments of the elastic potential energy ${\rm{\Delta }}E=\{{\rm{\Delta }}{E}_{p}^{1,c},\ldots ,{\rm{\Delta }}{E}_{p}^{k,c},\ldots ,{\rm{\Delta }}{E}_{p}^{K,c}\}$ can be given, where ${\rm{\Delta }}{E}_{p}^{1,c} > \ldots  > {\rm{\Delta }}{E}_{p}^{k,c} > \ldots  > {\rm{\Delta }}{E}_{p}^{K,c}$. Accordingly, the sequential set of edges, $e=\{{e}_{i,j}^{1},\ldots ,{e}_{i,j}^{k},\ldots ,{e}_{i,j}^{K}\}$, can be obtained, here, *i* ϵ *C*_*k*_ and *j* ϵ *C*_*c*,*c*_ (or *C*’_*c*,*c*_, the critical giant component of the modified network). Undoubtedly, the first element in the set of edges, ${e}_{i,j}^{1}$, is an optimal edge which, if added into the network, would improve the resilience of network maximally. The sequential set of optimal edges can be obtained naturally by repeating the above procedure. In this regard, a highly scalable algorithm, PA, is proposed for maximizing resilience. The algorithm is terminated if the number of added edges reaches a predefined limit, Fig. 3 shows the overall flowchart of the algorithm (more detailed depiction of the PA algorithm is shown in Supplementary Information section S5). Naturally, by adding the edges from the optimal set sequentially, the resilience of network can be enhanced maximally.

The resilience-improvement algorithm scales as $O(2\alpha K(M\,+\,N){\rm{l}}{\rm{o}}{\rm{g}}(M\,+\,N))$, where *M* is the number of edges of the network, *α*(*α«N*) is the number of pre-set optimal edges and *K*(*K«M*) is the number of large finite components (more detailed explanation in Supplementary Information section S5**)**. Generally, the number of large finite components *K* in a collapsed network is small, because the size distribution of the finite components follows the power law at the tail^29^. This high scalability allows us to find the edges to enhance the network resilience optimally in large-scale networks.

## Results

### Effectiveness

**Figure 4:**
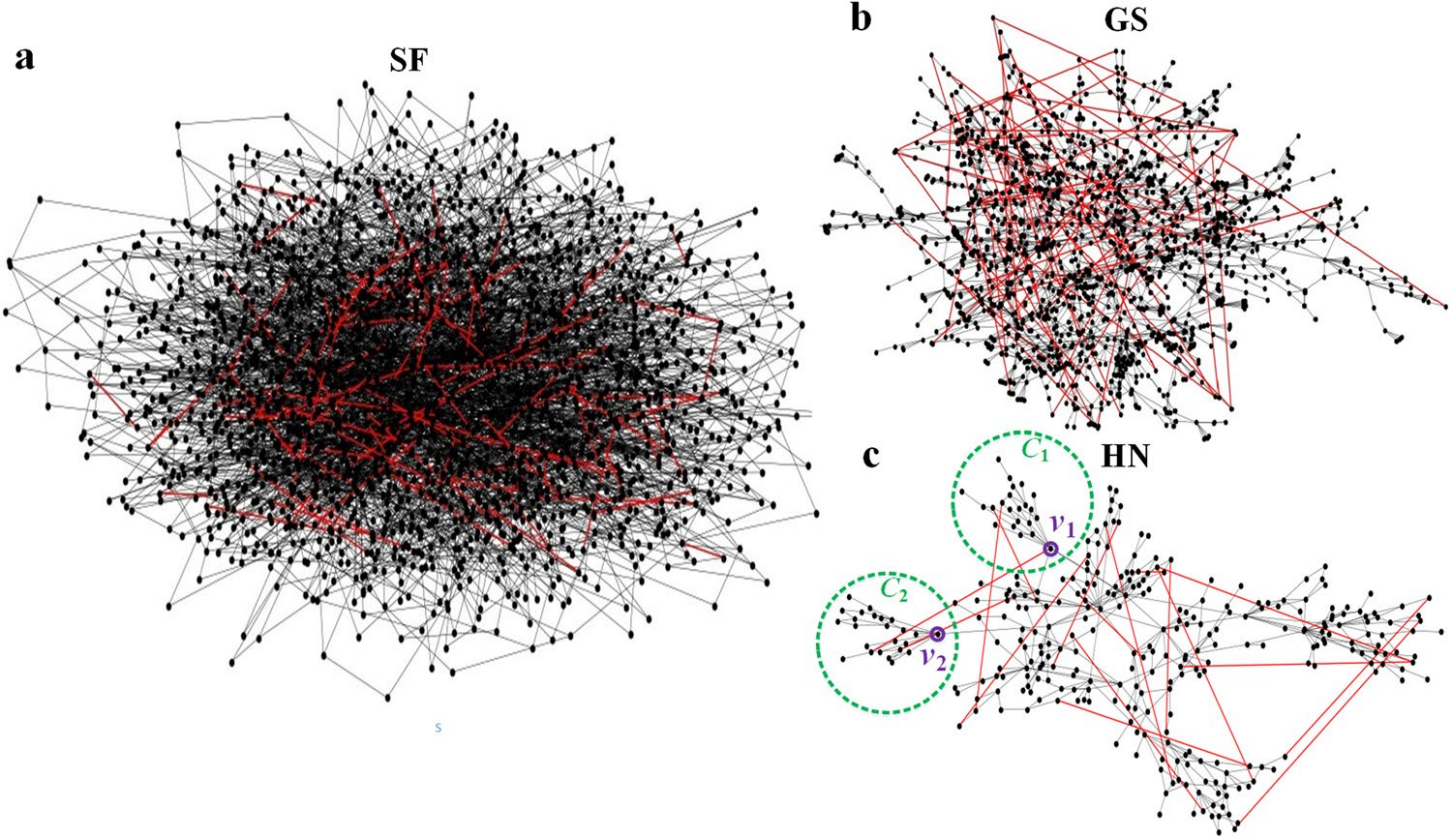
The optimized network structures. (**a**) The random SF network with *N* = 2000 nodes, *M* = 4000 edges, and power-law index *γ* = 3. (**b**) The GS power grid with *N* = 1569 nodes and *M* = 2163 edges. (**c**) The HN power grid with *N* = 310 nodes and *M* = 466 edges. In all cases, the test networks are modified by adding optimal edges (red), and the proportion of added edges to all edges of the original networks is 3.5%.

We demonstrate the efficiency of our approach on the Zachary (Karate club) network^26^, the Gansu (GS)^27^ and Henan (HN) power grids^28^ as well as artificial random networks, i.e., scale-free (SF) networks and Erdös-Rényi (ER) networks. Figure 1d–f demonstrates the effectiveness of the proposed algorithm in maximizing the resilience of a simple network (Zachary network^26^) against malicious attack (high degree adaptive, HDA). The network resilience is increased by 30%, 63% and 72%, by adding one, two and three edges, respectively. Figure 4a–c shows the structures of SF, GS and HN network optimized by the proposed method (the structure of the optimized ER network in Fig. S2). For example, in Fig. 4c, before optimization, the finite components (green) *C*_1_, *C*_2_ will emerge if the vital nodes (such as high degree nodes (purple)) *v*_1_, *v*_2_ are maliciously removed from original network; after being optimized by adding optimal edges (red), the emergence of *C*_1_, *C*_2_ will be avoided naturally under the same attacks. This case explains why the proposed method can tremendously improve network resilience. As a practical example, the networked micro grids can enhance the power system resilience^5^.

**Figure 5:**
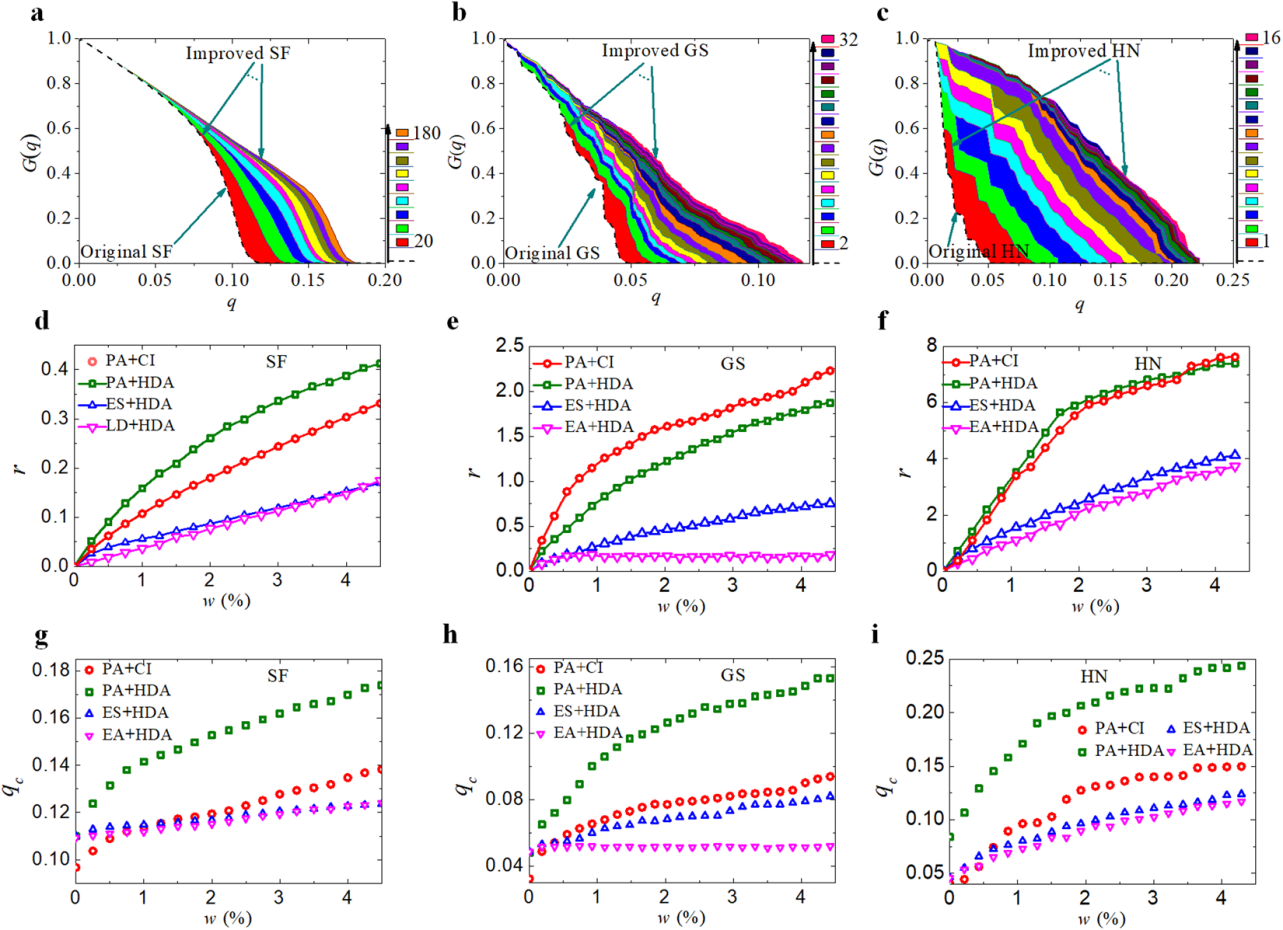
Mitigation against malicious attacks, improved resilience and critical threshold. (**a**–**c**) Mitigation against malicious attacks. The dashed lines correspond to the sizes of the giant components in each original network, the coloured solid lines to optimal modified networks under the different numbers of added edges and the coloured areas give the mitigation against malicious attacks (resilient increment). We compare the ratios (*r* = Δ*E*_*p*_/*E*_*p*_) of increased resilience of our algorithm (PA) with other methods (ES, LD) under two modes of malicious attacks (HAD and CI) for each network in (**d**–**f**). The abscissa, *w*, indicates the proportion of added (or swapped in the ES method) edges to all edges of the original networks. Here CI represents CI_2_ (other ratios of increased resilience and critical thresholds by CI_1_, CI_2_, CI_3_ and CI_4_ attacks are shown in Figs S4 and S5). The related comparisons of critical threshold increases for each network are shown in (**g**–**i**).

In Fig. 5a–c, we show the mitigation of malicious attacks for the SF network, GS and HN power grids (ER network in Fig. S3), respectively. The dashed lines correspond to the sizes of the giant component *G*(*p*) in each original network, and the coloured solid lines correspond to the typical modified networks under the different numbers of added edges (from 20 to 180, 2 to 32 and 1 to 16 for SF, GS and HN, respectively). The coloured areas give increments of the resilience (elastic potential energy) under malicious attacks. By adding only 4.5% of edges to the SF network, GS and HN power grids under HDA attacks (Fig. 5d–f), the resilience of the three networks were increased by 44%, 187% and 740%, respectively.

We compare the proposed algorithm with the heuristic strategies, i.e., ES^17^ and EA^23^ in Fig. 5d–f. Remarkably, the heuristic strategies (ES and EA) improve the network resilience greatly. Furthermore, the improvement ratios of the network resilience by our algorithm are the optimal ratios and are greater than those of the heuristic strategies^17,23^ under the same proportion of added (or swapped) edges. In the same three figures, we investigate the effect of the resilience improvement of our algorithm on two different malicious attacks, i.e., the widely used HDA^3^ and the optimal collective influence (CI)^12^ **(**see also Figs S4 and S5**)**. Our algorithm performs very well under both attacks. The network resilience is improved by 36%, 223% and 762% (by adding or swapping 4.5% edges) in the SF network, GS and HN power grids, respectively, under the CI attack. Furthermore, if the critical threshold is used as the resilience measure, our algorithm also outperforms the other strategies^17,23^ (Fig. 5g–i).

For networks with a community structure^29^ (such as the Zachary network, the GS and HN power grids), our algorithm produces a better network resilience and greater critical threshold than those complex networks with no community structure (such as SF and ER networks), as shown in Figs 5 and S3, because the networks lead to a few large finite components when they are attacked maliciously. In addition, better improvements of network resilience and critical percolation threshold can be obtained in the SF network (Fig. 5) than in the ER network (Fig. S3). As the top vital (hub) nodes of the SF network are removed sequentially, its serious heterogeneity will generate a few large finite components, which contributes to the consequences. Figure S6 shows that the network resilience and the critical thresholds of the original and the improved ER networks are increased, which indicates that they follow nearly the same rising trend in the original and the improved networks as the average degree. From Fig. S7, one can observe that the improvements in the network resilience and the critical threshold remain nearly unchanged regardless of the network size.

### Unchanged network functionality

**Figure 6:**
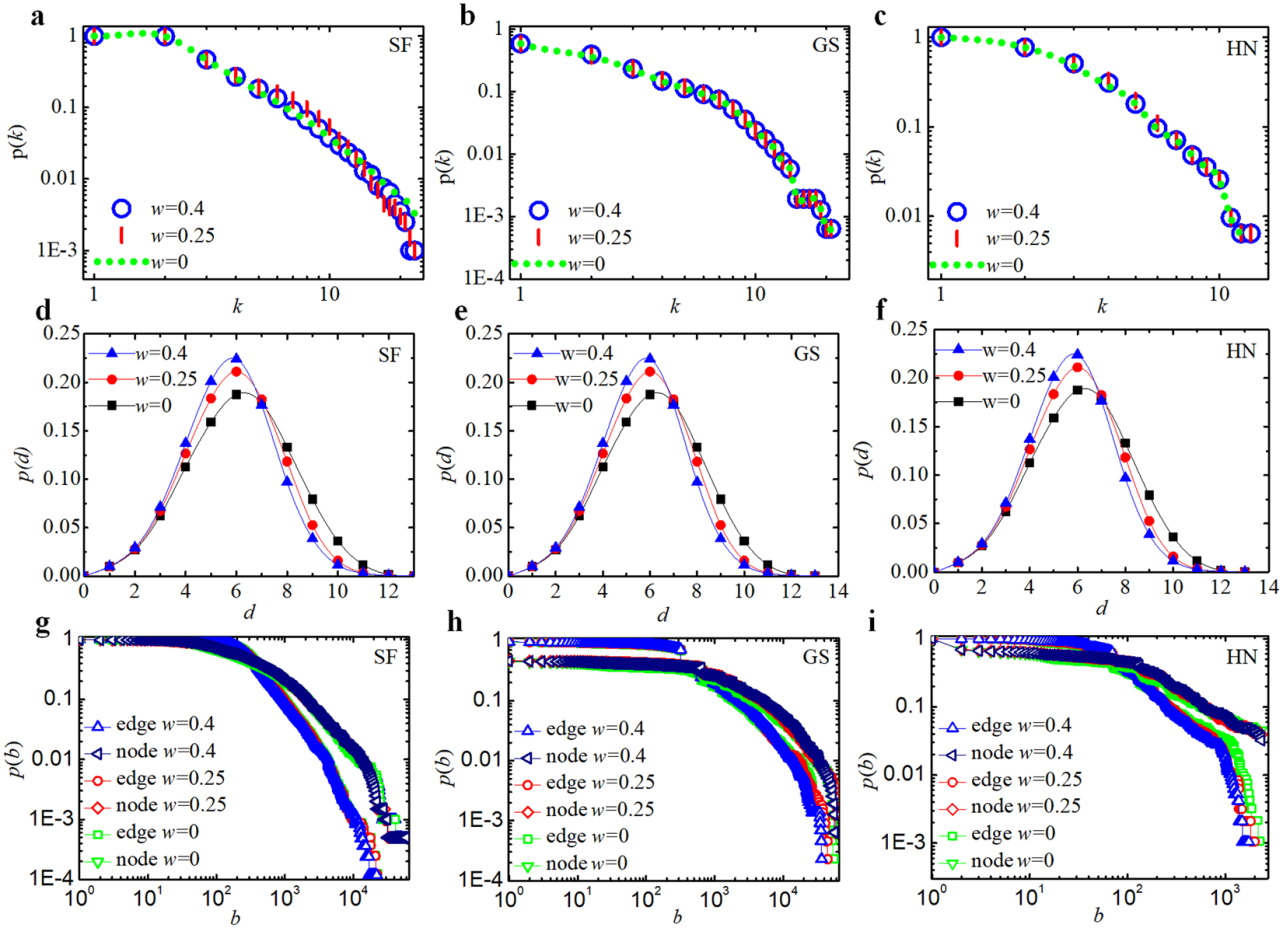
Unchanged network functionality. The network functionality is characterized by the network topological structure. The test networks with *w* = 0.25 and *w* = 0.4 were modified by our algorithm based on HDA attacks; the networks with *w* = 0 denote the original networks. (**a**–**c**) The cumulative degree distribution *p*(*k*). (**d**–**f**) The cumulative shortest path distance distribution *p*(*d*), where *d* is the shortest path distance between nodes. (**g**–**i**) The cumulative between-ness distribution *p*(*b*), where *b* represents between-ness of node or edge.

The functionality of a network is commonly related to its topological features^17,29^. It is fundamental and necessary to keep a network’s functionality unchanged when optimizing its resilience. We tested the effects of the topological structural changes on the functionalities of the optimized networks, i.e., the SF network, and the GS and HN power grids. The distributions of cumulative degree, shortest path distance and betweenness were used for measuring the functionality. As shown in Fig. 6, those functionality measures hardly changed. Other topological characteristics including the cluster coefficient, the network diameter, *etc*., also remain unchanged (Table S2). Therefore, the networks optimized by our algorithm are not only more resilient against malicious attacks but also exhibit little change to their functionalities compared with the original networks.

## Discussion and Conclusion

Intentional attacks and the corresponding defences are always the two opposite sides of network security. To enhance network resilience against malicious attacks, we introduce the network resilience indices by mapping a complex network onto a physical elastic system; then we propose a unified theoretical framework and a general approach (PA algorithm) to solve the problem of resilient optimization. As mentioned before, both the ES methods and EA methods cannot well maintain the topological functionality of a network and their performance on resilience improvement cannot be guaranteed since they are unable to optimize network resilience globally under a theoretical framework. In contrast, our algorithm can maximize network resilience by adding optimal edges between the “weak cores” and the critical giant component (Fig. 1), with minimal costs. This is because, after being optimized by our method, the emergences of the large infinite components can effectively be avoided under the same attacks (Figs 1 and 4). Moreover, the proposed indices of network resilience can characterize the elastic properties for nonlinear networks, compared with the conventional metrics such as critical threshold. Case studies show that our algorithm achieves better performance on resilient improvement of networks, compared with competing approaches^17,23^.

As edges are added to reach a certain proportion, the growth of network resilience slows down, especially for realistic networks, because the number of large-scale finite components generated by malicious attacks becomes increasingly smaller. Thus, it is necessary to balance the maximum resilience improvements with the costs of modifying a network to find an optimal compromise for the application of our method.

The proposed theory is strictly valid, and can be applied to any real network. Our solution to the optimal resilience problem demonstrates its importance because it can be used to enhance network resilience, guide the design of technological resilient systems, and offer fast and effective ways to mitigate the collapse of networks against malicious attacks, or furnish a self-healing solution to reconstruct existing failed infrastructure systems.

## Supplementary information

Supplementary information
