# Supplementary material for: Maximizing Network Resilience against Malicious Attacks
Source: Sci Rep. 2019 Feb 19;9:2261. doi: 10.1038/s41598-019-38781-7 (PMC6381220; doi:10.1038/s41598-019-38781-7)
Supplement: Supplementary file 1 — Supplementary information [file 41598_2019_38781_MOESM1_ESM.pdf]

Maximizing Network Resilience against Malicious Attacks
(Supplementary information)

Wenguo Li [1, 2, +], Yong Li [1, *, +], Yi Tan [1, *], Yijia Cao [1, +], Chun Chen [1], Ye Cai [3], Kwang Y. Lee [4] and Michael Pecht [5]

[1]College of Electrical and Information Engineering, Hunan University, Changsha 410082, China.
[2]School of Information and Electronic Engineering, Hunan City University, Yiyang 413000, China.
[3]School of Electrical and Information Engineering, Changsha University of Science and Technology, China, Changsha 413000, China.
[4]Department of Electrical and Computer Engineering, Baylor University, Waco, Texas 76798-7356, USA.
[5]CALCE Electronics Products and Systems Center, University of Maryland, College Park, Maryland 20742, USA.
*Correspondence authors: yongli@hnu.edu.cn (Y.L.) and yibirthday@126.com (Y.T.).
[+]These authors contributed equally to this work.

S1 Network resilience indices

Network resilience is a useful concept, and several research achievements have been reported recently in defining resilience and the assessment of networks[1-3]; however, there are no universally accepted indices and definition of network resilience[4]. Here, we map a network resilience system onto a physical elastic system and propose a resilience analysis framework and a metric for measuring network resilience. Based on the physical elastic system, we define resilience-relative concepts and indices of networks, as shown in **Fig. S1a** and **Table S1**. Network resilience refers to the deformation of a network under the action of external forces that can restore its original size and shape if the external force is withdrawn. As shown in **Table S1**, the external force on a network is the fraction of vertices removed initially, q, and the elastic deformation of a network denotes the fraction of failed vertices, 1-$G(q)$, due to the external forces (perturbations or attacks), where $G(q)$ is the largest (giant) connected-component fraction of the network.

In analogy with a physical elastic system, the resilience indices of a network system include an elastic coefficient (also called the modulus of elasticity or Young's modulus) K, a critical percolation threshold, q_c (or the critical elastic deformation, 1-$G(q_c)$) from a statics point of view, and the elastic potential energy, E_p (or elastic complementary energy, E_c) in the view of energy. For the elastic coefficient of a network, a normal of the stress-strain (giant component) curve in the elastic region satisfies the Hooke's law, given by

$$K = \frac{\mathrm{d}F}{\mathrm{d}\sigma} = \frac{\mathrm{d}q}{\mathrm{d}(1-G(q))} \tag{S1}$$

where F is elastic force (external force, q), σ is the elastic deformation 1-$G(q)$, and $K \in [0,1]$. The tangent to the giant component curve is

$$T = \frac{\mathrm{d}G(p)}{\mathrm{d}p} \tag{S2}$$

Since the elastic coefficient, K, equals the negative reciprocal of the tangent, T, as shown in **Fig.**

S1a, the elastic coefficient of network can be obtained from equations (S1) and (S2) by

$$K = -\frac{1}{T} = -\frac{1}{\frac{\mathrm{d}G(q)}{\mathrm{d}q}} \tag{S3}$$

For a network resilience system, the existence of a giant component means that the network is within the elastic deformation limit and is capable of recovering from failures. The condition of existence of the giant component is defined as[5]

$$K = \frac{\langle k^2 \rangle}{\langle k \rangle} \tag{S4}$$

where k is the vertex degree, and averages, indicated by angular brackets, are taken over all of the vertices of the network. From equation (S4), the critical threshold (critical external forces) of percolation, q_c, can be obtained by [5]

$$q_c = 1 - \frac{1}{K_0 - 1} \tag{S5}$$

Table S1. The mapping relation between a **resilient network and a physical elastic system.** Here, $G(q)$ is the largest (giant) connected component fraction of the network and is also a function of the fraction q of removed vertices.

Concepts and indices	Physical elastic system	Resilient network
External force	F	q (The fraction of removed nodes)
Elastic deformation	σ	1-$G(q)$ (The fraction of failed vertices)
Critical external force	F_c	q_c (The critical percolation threshold)
Critical elastic deformation	σ_c	1-$G(q_c)$
Elastic coefficient	$K = \mathrm{d}F / \mathrm{d}\sigma$	$\mathrm{d}q / \mathrm{d}(1-G(q))$
Condition of elastic limit	$F = F_c$ or $\sigma = \sigma_c$	$K = \frac{\langle k^2 \rangle}{\langle k \rangle} = 2$
Elastic complementary energy	$E_c = \int_{F=0}^{F=F_c} \sigma \mathrm{d}F$	$E_c = \int_{q=0}^{q_c} (1 - G(q))\mathrm{d}q$
Elastic potential energy	$E_p = \int_{\sigma=0}^{\sigma_c} \text{-}F\mathrm{d}\sigma = 1\text{-}E_c$	$E_p = \int_{1\text{-}G(q)=0}^{1\text{-}G(q_c)} \text{-}q\mathrm{d}(1\text{-}G(q)) = 1\text{-}E_c$

where $K_0 = \frac{\langle k_0^2 \rangle}{\langle k_0 \rangle}$ is calculated using the original distribution before the vertices are removed. Correspondingly, we can acquire the elastic deformation limit (critical elastic deformation, $(1 - G(q))$), which is equivalent to the critical threshold (critical external force, q_c) for a measure of

network resilience.

Based on original definition of a physical elastic system, the elastic complementary energy and the elastic potential energy of networks can be respectively defined by (**Fig. S1a**)

$$E_c = \int_{F=0}^{F=F_c} \sigma \mathrm{d}F = \int_{q=0}^{q=1} (1-G(q))\mathrm{d}q \tag{S6}$$

$$E_p = \int_{1\text{-}G(q)=0}^{1\text{-}G(q_c)} \text{-}q\mathrm{d}(1\text{-}G(q)) = \int_{1\text{-}G(q)=0}^{1\text{-}G(q_c)} q\mathrm{d}G(q) = \int_{q=0}^{q=1} G(q))\mathrm{d}q \tag{S7}$$

And the elastic complementary energy and the elastic potential energy of networks can be obtain by

$$E_c + E_p = 1 \tag{S8}$$

The numerical versions of equation (2) by rectangular and trapezoid approximation method respectively, are given in the main text, i.e., equations (3) and (4) in main text. The numerical integration error of the former is $|R_r| = \frac{h(b-a)}{2} = \frac{1}{2N}$, where $h=1/N$ is the minimum-step integral size, (a, b) is the integrating range, where a = 0, b = 1; the error of the latter is $|R_t| = \frac{(b-a)}{12} h^2 M = \frac{1}{12N^2}$, where M = 1 is upper limit. Hence, the latter is smaller than former normally.

In linear physical systems, external force limit, elastic deformation limit and the elasticity coefficient (K) are generally used to measure resilience. However, for a nonlinear network system, the elasticity coefficient (K) varies nonlinearly with the external force (critical threshold) (q), and there is non-linear relationship between external force and elastic deformation. The above three resilient indices can only characterize the network resilience to some degree; however, it may not be useful in many realistic cases. Those measure, for example, ignores magnitude of elastic deformation in the elastic limit. The elastic potential energy can characterize its elastic properties better due to its advantages covering the elastic coefficient and critical threshold, as shown in **Fig. S1b**.

S2. Method to identify vital nodes for malicious attacks

In this section we describe the existing algorithms in the literature to identify vital nodes that have been used to intentionally attack complex networks. Those algorithms are the bases on which we tested the performance of our algorithm for optimal network resilience. Real networks exhibit heterogeneous natures with nodes playing different roles in structure and functionality. There are numerous heuristic identification algorithms ranging from simply counting the immediate neighbours to complicated machine-learning and message-passing approaches. Here we use two methods as follows:

High Degree Adaptive (HDA) Method[6]. The HDA method is a typical malicious-attack algorithm in which nodes are ranked by degree and sequentially removed, starting with the node of the highest degree. Note that in the HDA method, the degrees of the remaining nodes are recomputed after each node removal. One characteristic of this method is the fact that hubs may form tightly

knit groups called "rich-clubs". Strategies based on high degree will rank these rich-clubs highly. The HDA algorithm is more effective than the High-Degree (HD)[7] algorithm due to its avoiding the areas already attacked in the network.

Collective Influence (CI) Method[8]. This is an optimal malicious-attack algorithm. In the CI algorithm, Morone and Makse introduce the concept of collective influence, which is the product of a node's reduced degree (the number of its nearest connections minus one) and the total reduced degrees of the nodes. By taking the collective influence effects into account, CI optimization theory identifies a new class of strategic influencers called "weak nodes", which outrank the hubs in the network. If the high-collective-influence nodes are removed sequentially, the networks would break down into many disconnected components; as a result, the network undergoes a structural collapse at a critical point. Naturally, this algorithm is more effective in fragmenting a network than removing the hubs or nodes identified by other algorithms such as PageRank or *K*-core. In addition, the CI algorithm is remarkable for its advantage of computational complexity because it requires only $N^2\log N$ computation. This high scalability allows us to use this algorithm to find top influencers in large networks.

S3. Heuristic methods to enhance resilience in complex networks

The heuristic methods of improving resilience (or robustness) are described in this section. We compare these heuristics with our theory of maximization of network resilience. A common feature of the heuristic methods is that they improve network resilience by modifying topological structure; they are not designed from first principles.

Edge Addition (EA) Method[9]. One possible way to enhance the resilience of a network is to add new edges to it. Ref. 9 introduces an EA algorithm to guide the resilience of scale-free (SF) networks. In the **EA** algorithm, for a given network, the pairs of independent nodes with lowest degrees are selected and new edges are established between these node pairs. The EA algorithm can enforce network resilience to some degree; however, it is not optimal. This algorithm scales as $\sim O(N)$, where N is the number of vertices of the whole network.

Edge Swap (ES) Method[6]. Instead of considering the criterion of critical threshold q_c for network resilience (robustness), Schneider, *et al*. introduce an index R for the robustness of a network; R is defined as

$$R = \frac{1}{N}\sum_{q=\frac{1}{N}}^{1} G(p) \qquad \text{(S9)}$$

where $G(q)$ is the fraction of vertices in the giant component after removing qN nodes and $1/N$ is a normalization factor that makes it easier to compare the robustness of networks with different sizes. The heuristic algorithm for maximizing R with the nodal degree fixed is to pick random pairs of edges e_{ij}, e_{kl} and swap them to e_{il}, e_{jk}, in which case the robustness increases significantly only if $R_{new} > R_{old} + \delta_R$, where δ is a threshold value. Note that the threshold δ_R should be set so large that nearly every change is rejected. Therefore, the ES algorithm is a greedy method and is a near-optimal solution. The networks optimized by the ES algorithm have an onion-like structure in which high nodes form a core with radially decreasing degrees and over-presentation of edges within the same radial layer. Since the swapping of two arbitrary edges can impact the value of R, the complexity of swapping all sets of two arbitrary edges scales as $O(M^2)$; all in all, the computational

complexity of the traditional methods[6] is $O(\omega M^2(M+N)\log(M+N))$, where ω is the number of swapped edge pairs. Hence, high runtime makes the heuristic algorithms prohibitively slow for large networks.

S4. Theoretical analysis of incremental resilience

For cases (i) and (ii) in the main text, the two percolation curves of the giant components in the original and modified networks, $G_O(q)$ and $G_I(q)$ (explained in Fig. 2a), almost coincide (i.e., $G_O(q) \approx G_I(q)$ for $q \in [0, q_c]$).The increment of elastic potential energy for the two cases (Fig. 2a in the main text) is

$$\Delta E_p = \int_0^{q_c} G_I(p) - \int_0^{q_c} G_I(p) \approx 0 \tag{S10}$$

Suppose that the two finite components, C_a and C_b, fail at q_a and q_b ($q_a < q_b$), respectively. In case (iii) of the main text, the edge e_{ij} added between their "weak cores", i.e., $C_{a,c}$ and $C_{b,c}$, mitigates the failure of the finite component C_a at q_a until the finite component C_b fails at q_b (Fig. 2 b in main text). Accordingly, the increment of elastic potential energy in this case can be obtained by

$$\Delta E_p^{a,b} = \int_{q=0}^{q=1} [G_I(q) - G_O(q)]\mathrm{d}q = \int_{q_a}^{q_b} [G_I(q) - G_O(q)]\mathrm{d}q \tag{S11}$$

where $G_I(q) = G_O(q)$ for $q \in [0, q_a] \cup [q_b, q_c]$.

When an edge e_{ij} is added between the "weak cores" $C_{a,c}$ and the critical giant component $C_{c,c}$ in case (iv) of the main text, the increment of elastic potential energy (Fig. 2c in the main text) is given by

$$\Delta E_p^{a,c} = \int_{q=0}^{q=1} [G_I(q) - G_O(q)]\mathrm{d}q = \int_{q_a}^{q_c} [G_I(q) - G_O(q)]\mathrm{d}q + \int_{q_c}^{q_{cI}} G_I(q) \tag{S12}$$

where $G_I(q) = G_O(q)$ for $q \in [0, q_a]$, q_{cI} is the critical threshold of the modified network.

Since $G_I(q) > G_O(q)$ for $q \in [q_a, q_b]$ in case (iii) of the main text and $G_I(q) > G_O(q)$ for $q \in [q_a, q_c]$ in case (iv) of the main text, the left sides of equations (S11) and (S12) are greater than 0, i.e., $\Delta E_p^{a,b} > 0$ and $\Delta E_p^{a,c} > 0$. Comparing (S10) with equations (S11) and (S12), the difference between the increments of elastic potential energies in cases (i) and (ii) of the main text is less than that between cases (iii) and (iv) of the main text.

Comparing equation (S11) with equation (S12), the difference between the increments of elastic potential energies in cases (iv) and (iii) of the main text can be obtained by

$$\Delta E_p^{a,c} \text{-} \Delta E_p^{a,b} = \int_{q_c}^{q_{cI}} G_I(q)\mathrm{d}q + \int_{q_b}^{q_c} [G_I(q) - G_O(q)]\mathrm{d}q \tag{S13}$$

where $\int_{q_a}^{q_c} [G_I(q) - G_O(q)]\mathrm{d}q = [\int_{q_b}^{q_c} G_I(q)\mathrm{d}q + \int_{q_a}^{q_b} G_I(q)\mathrm{d}q] + [\int_{q_b}^{q_c} G_O(q)\mathrm{d}q + \int_{q_a}^{q_b} G_O(q)\mathrm{d}q]$.

The left side of equation (S13) must be larger than 0 (i.e., $\Delta E_p^{a,c} \text{-} \Delta E_p^{a,b} > 0$) because $q_c > q_b$ and $G_I(q) > G_O(q) \geq 0$ for $q \in [q_b, q_c]$, i.e., the increment of elastic potential energy in case (iv) of the main text is greater than that in case (iii). Therefore, the optimal edge, e_{ij}, must be located

between a “weak core” and the critical giant component (i.e., case (iv)).

S5. Detailed description of the PA algorithm

We develop a method for modifying topology, i.e., a **Posteriorly Adding** (**PA**) edges algorithm to maximize network resilience against malicious attacks, as shown in Fig. 1 of the main text. The related theory of the algorithm is shown in the main text (equations (1)-(4)) and in Supplementary Information Section S4 (equations (10-14)). Here, we present the proposed **PA** algorithm in more detail as follows:

Step 1. Maliciously attack the original network until it breaks down (the critical giant component $C_{c,c}$ appears). In the process, search the connected graphs, including the giant component and finite components, using the breadth-first search (BSF) algorithm, store the size s and failed sequence q for every finite component and the critical threshold q_c, and calculate the elastic potential energy of the original network according to (3) in the main text.

Step 2. Search the “weak cores” ($C_{k,c}$, the critical giant component of finite component C_k) for every finite component whose size is greater than 1 percent of the original network and construct potential edge set $\mathbf{e}=\{e_{i,j}^{k} \mid i\in C_{k,c}, j\in C_{c,c}\}$ between each ‘weak core’ and the critical giant component.

Step 3. Compute the potential increment of elastic potential energy of each edge $e_{i,j}^{k}$ from (S12) independently. After tentatively adding the edge into the original network and attacking the modified network as in **Step 1**, obtain the sequential optimal edge set $\mathbf{e}=\{e_{i,j}^{1},...,e_{i,j}^{k},...,e_{i,j}^{K}\}$ by the sizes of their increments of elastic potential energy, where K is the number of edges (or “weak cores”).

Step 4. Add a new edge $e_{i,j}^{1}$ from the sequential optimal edge set **e**, and compute the potential increment of the added edge

Step 5. Modify the network structure by adding the optimal edges $e_{i,j}^{1}$.

Step 6. Repeat **Steps 1-5**.

Terminating condition: the number of added edges reaches the pre-set number.

The time complexity from **Step 1** to **Step 2** is $O((M+N)\log(M+N))$ for searching the giant component, the finite components and the “weak cores”, where M and N are, respectively, the numbers of edges and vertices of the whole network. The runtime of **Step 3** is $O(K(M+N)\log(M+N))$ because **Step 1** is repeated K times in **Step 3**, where K is the number of the “weak cores” in **Step 2**.. In **Step 6 (Steps 1-5)** are repeated *α times*, where α is the number of the pre-set optimal edges. Therefore, the proposed PA algorithm scales as $O(\alpha K(M+N)\log(M+N))$, where $\alpha \ll M$ and $K \ll M$. The time complexity of the PA algorithm is near that of the EA [9] algorithm and far less than that of the ES[6] algorithm. Note that the nodes v_i, v_j, i.e., the terminal nodes of the added edge e_{ij}, should be selected as the least important nodes in the “weak cores” and giant component.

Table S2. Topological characteristics of networks. The topological characteristics include the cluster coefficient (*C*), network diameter (*D*), average of shortest-path distance <*L*>, topological entropy (*E*) and degree-correlation coefficient (*CC*). The networks with w = 0.2 and w = 0.4 (SF, GS and SF) were modified by our algorithm based on HDA attacks and the networks with w = 0 denote the original networks. The SF network is composed of N = 2000 nodes, M = 4000 edges, average degree <*k*> = 4.

Networks		*C*	*D*	<*L*>	*E*	*CC*
GS [27]	*w*=0	0.190319	25	9.932539	0.88985	2.678629
	w=0.25	0.186153	20	8.529675	0.890419	2.55363
	w=0.4	0.18219	18	7.17067	0.89176	2.589768
HN [28]	*w*=0	0.158773	13	6.171417	0.903649	1.650535
	w=0.25	0.149741	13	5.833824	0.906066	1.595849
	w=0.4	0.232038	13	4.728778	0.826045	4.740009
SF	*w*=0	0.024207	8	4.164414	0.849916	-0.46805
	w=0.25	0.023814	8	4.153477	0.84766	-0.465701
	w=0.4	0.025847	7	4.067881	0.84766	-0.479959

S6. Supplementary figures

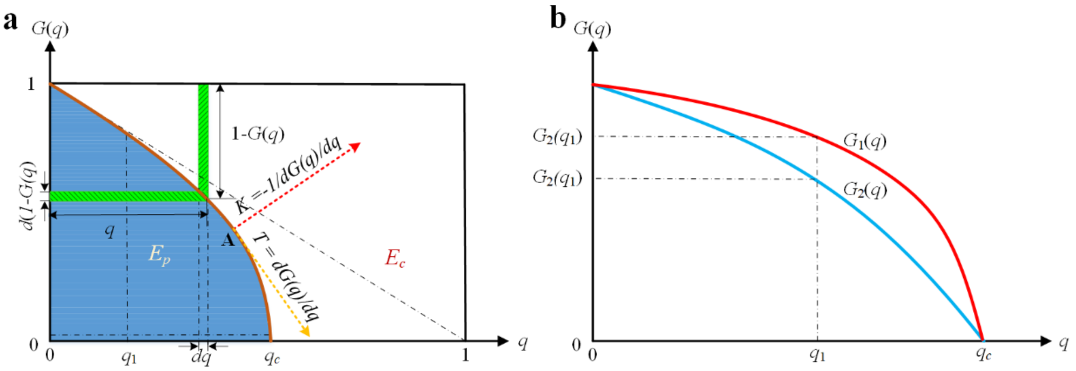

Figure S1. The indices and resilience-related concepts of a network system. a. T is a tangent to the giant component curve, and the elastic coefficient of the network, K, is the normal to the giant component curve at q, which equals the negative reciprocal of T at q. **b**. Comparison of two network resilience indices, i.e., the elastic potential energy and elastic limit. The elastic limit equals external force limit (the critical threshold, q_c), and its effect is equivalent to elastic deformation limit, $1\text{-}G(q)$.

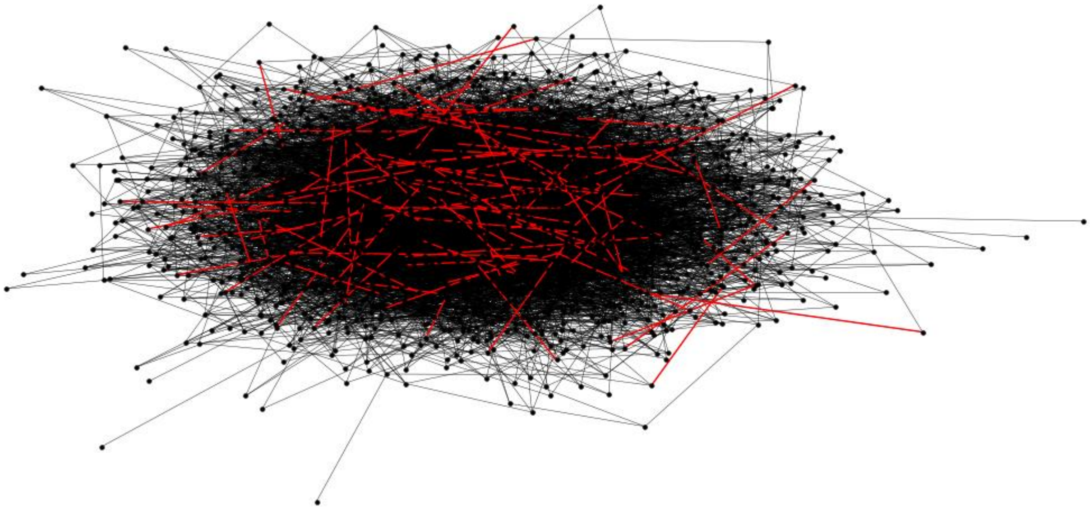

Figure S2. The structure of optimized ER network. The ER network with N = 2000 nodes, M = 16000 edges, is modified by adding optimal edges (red), and the proportion of added edges to all edges of the original networks is 2%.

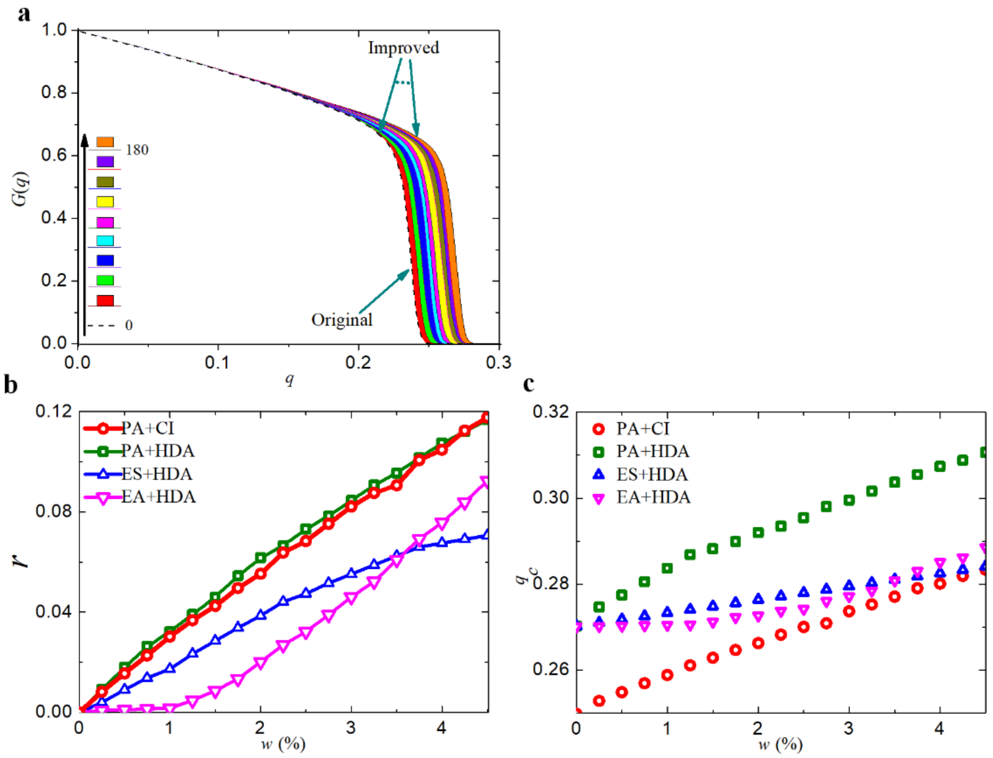

Figure S3. Mitigation of malicious attacks, improved resilience and critical threshold of ER network. The ER network is composed of N = 2000 nodes, M = 4000 edges. **a**. The mitigation of malicious attacks. The dashed lines correspond to the size of the giant component in each original network, the coloured solid lines to typical modified networks with different numbers of added edges (from 20 to 180), and the coloured areas give the mitigation of malicious attacks, namely, increments of resilience (elastic potential energy). We compare the ratios of resilience increases of our algorithm (PA) with those of other methods (ES, EA) under two malicious attacks (the typical HAD and the optimal CI) in **b**. The related comparison of the critical thresholds for each network is shown in **c**.

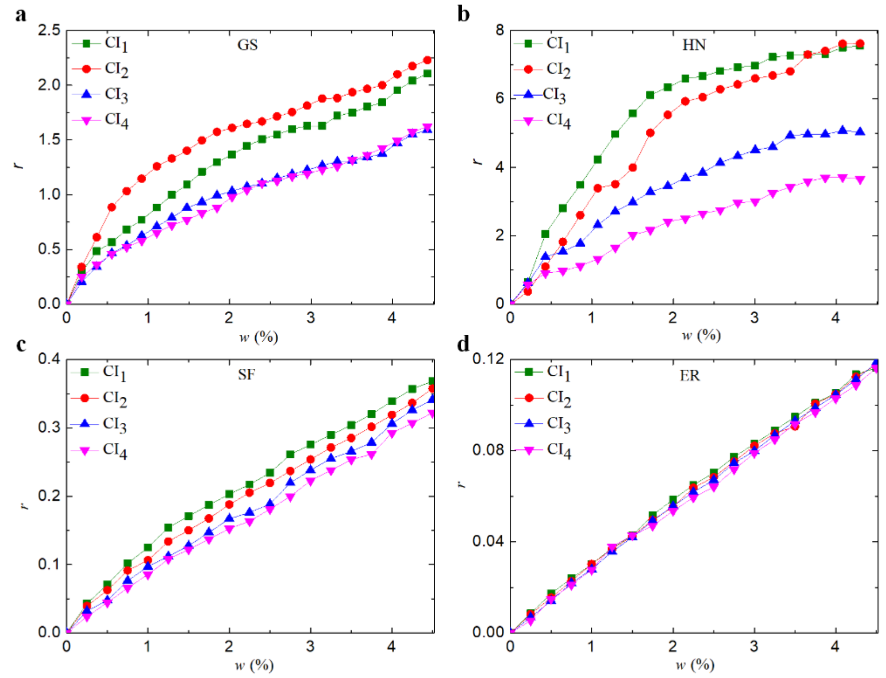

Figure S4. Ratio of increased resilience by our algorithm based on CI attacks. The ER and SF networks are composed of N = 2000 nodes, M = 4000 edges respectively. We plot the ratios of increased resilience by our algorithm plus CI_l attack algorithm with different parameters l (l = 1, 2, 3 and 4) for four networks (GS, HN, SF and ER) in **a**, **b, c** and **d.**

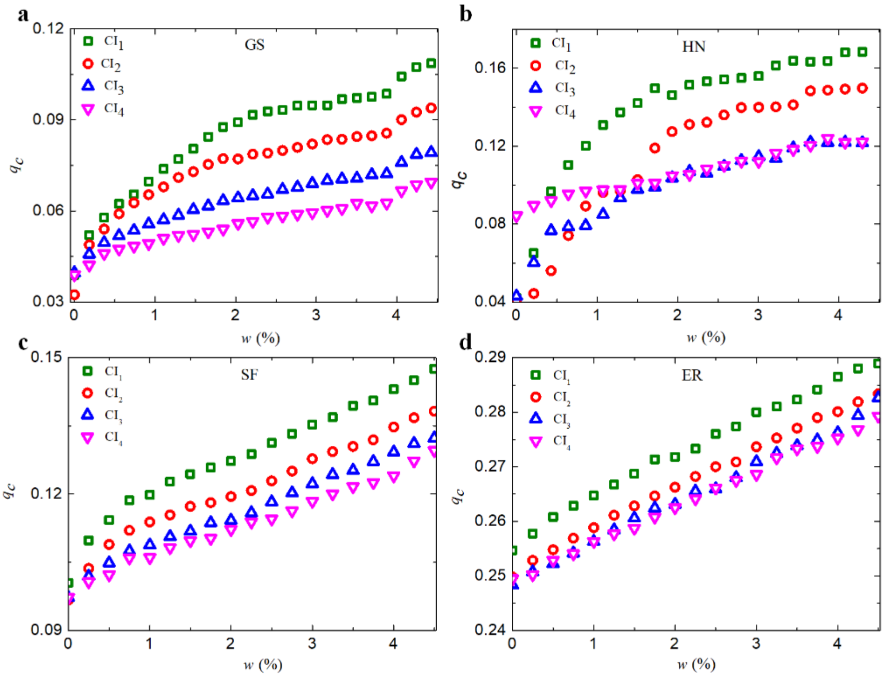

Figure S5. Increase of critical threshold by our algorithm against CI attacks. The ER and SF networks are composed of N = 2000 nodes, M = 4000 edges respectively. We show the increase of critical threshold by our algorithm plus the CI_l attack algorithm with different parameters l (l = 1, 2, 3 and 4) for four networks (GS, HN, SF and ER) in **a**, **b, c** and **d.**

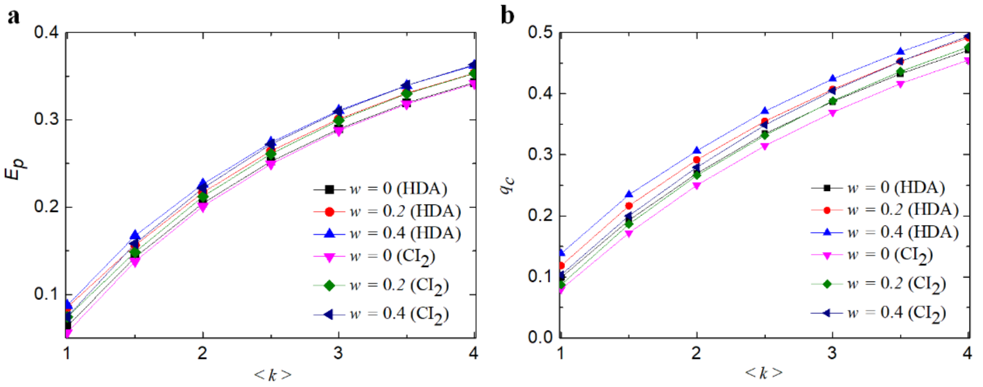

Figure S6. Resilience (elastic potential energy, E_p) and critical threshold versus average degree (<k>) in the ER network. The ER network is composed of N = 2000 nodes. In **a** and **b**, we show, respectively, the increases of the resilience and critical thresholds of our algorithm based on two attack modes (HDA and CI) for the ER network with different average degree <k>, where the network with w = 0 denotes the original ER network, and w = 0.2 and w = 0.4 represent the different modified ER networks.

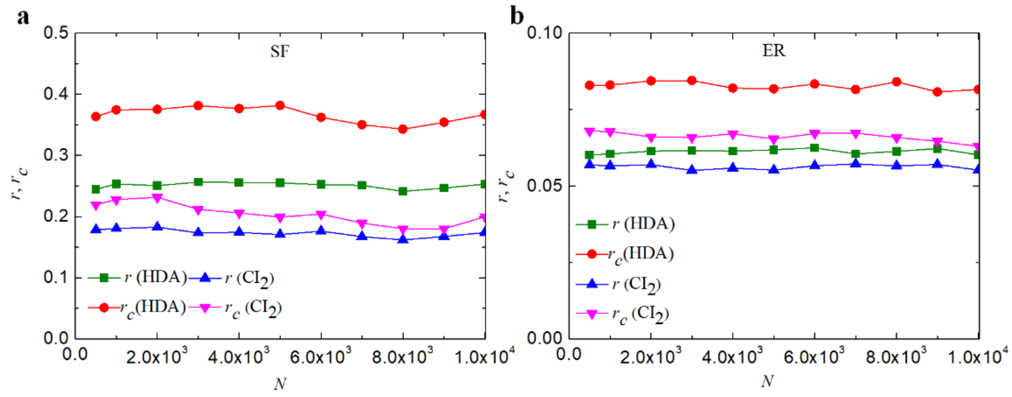

Figure S7. The ratio of resilience increase versus the size of networks. The ratios of resilience increase r (= ΔE_p / E_p) and critical threshold r_c (=$\Delta p_c/p_c$) versus network size N for (**a**) SF networks with N = 2000 nodes, M = 4000 edges, power-law index γ = 3, average degree <k> = 4 and (**b**) ER networks with N = 2000 nodes, M = 4000 edges, average degree <k> = 4.

S7. Supplementary references

1. Royce, A. F, Behailu, B. A metric and frameworks for resilience analysis of engineered and infrastructure systems. *Reliability Engineering and System Safety*, **121**, 90–103 (2014).
2. Commonwealth of Australia. Critical infrastructure resilience strategy. *Canberra: Commonwealth of Australia* (2010).
3. Vugrin, E. D, Warren, D. E, Ehlen, M. A. A resilience assessment framework for infrastructure and economic systems: quantitative and qualitative resilience analysis of petrochemical supply chains to a hurricane. *Process Safety Progress*, **30**, 280–90 (2011).

4. Li, Z., Shahidehpour, M., Aminifar, F., Alabdulwahab, A. Networked microgrids for enhancing the power system resilience. *Proceedings of IEEE*, **105**, 1289-1310 (2017).
5. Cohen, R., & Havlin, S. Complex networks: structure, stability and function. *Cambridge University Press*, Cambridge (2010).
6. Schneider, C. M.; Moreira, A. A., Andrade, J. S., Havlin, S., Herrmann, H. J. Mitigation of malicious attacks on networks. *Proc. Natl. Acad. Sci*. USA, **108**, 3838–3841 (2011).
7. Callaway, D. S., Newman, M. E. J., Strogatz, S. H., and Watts, D. J. Network robustness and fragility: Percolation on random graphs. *Phys. Rev. Lett*., **85**, 5468 (2000).
8. Morone, F. & Makse, H. A. Influence maximization in complex networks through optimal percolation. *Nature* **524**, 65-68 (2015).
9. Jiang, Z., Liang, M. & Guo, D. Enhancing network performance by edge addition. *Int. J. Mod. Phys. C* 22, 1211.9 (2011).
